# Gold nanoparticles – an optical biosensor for RNA quantification for cancer and neurologic disorders diagnosis

**DOI:** 10.2147/IJN.S181732

**Published:** 2018-11-29

**Authors:** Sherif M Shawky, Ahmed M Awad, Arwa A Abugable, Sherif F El-Khamisy

**Affiliations:** 1 Center for Genomics, Helmy Institute for Medical Sciences, Zewail City of Science and Technology, Giza, Egypt, s.el-khamisy@sheffield.ac.uk; 2 Krebs Institute, Department of Molecular Biology and Biotechnology, Firth Court, University of Sheffield, Sheffield, UK, s.el-khamisy@sheffield.ac.uk; 3 Biochemistry Department, Faculty of Pharmacy, Misr University for Science and Technology, Giza, Egypt; 4 Department of Molecular Biology, Genetic Engineering and Biotechnology Research Institute, University of Sadat City, Sadat City, Egypt

**Keywords:** gold aggregating gold, TOP1, TDP2, magnetic nanoparticles, RNA extraction, genomic instability, neurodegenerative diseases, tumor markers

## Abstract

**Purpose:**

The objective of this study is to develop a facile tool for the absolute detection and quantification of nucleic acid transcripts, using a gold nanoparticle-based optical biosensor. Topoisomerase 1 (TOP1) and tyrosyl DNA phosphodiesterase 2 (TDP2) were among the nucleic acid transcripts of choice due to their role as genomic instability biomarkers and their implication in various cancers and neurologic disorders. This opens the door to develop a simple tool that can be used for diagnosing and monitoring treatment response for such diseases, overcoming the requirements for high cost, time, and complexity of the existing technologies for the absolute quantification of transcripts of interest.

**Materials and methods:**

The TOP1 and TDP2 mRNA transcripts were first captured specifically using magnetic nanoparticles that were functionalized with TOP1- and TDP2-specific probes, respectively. The captured mRNA was then directly detected and quantified using the gold aggregating gold (GAG) assay, without the need for amplification as in existing technologies used for the quantification of transcripts.

**Results:**

A linear correlation exists between the GAG assay and the qPCR for the quantification of the TOP1 and TDP2 mRNA transcripts (10^1^–10^4^ copies). The detection limit of the GAG assay in mRNA quantification was up to 10 copies per reaction. Wild-type and TDP2-deficient cell lines confirmed the assay specificity and reproducibility in distinguishing between different transcripts.

**Conclusion:**

The GAG assay can be utilized as an inexpensive, rapid, simple, and sensitive tool for the absolute quantification of RNA for different applications, instead of the laborious, expensive, and sophisticated real-time PCR.

## Introduction

An efficient healthcare system is dependent on three main pillars: patients (disease), efficient and affordable diagnosis, as well as rapid and precise clinical decisions. Molecular diagnostics and particularly, nucleic acid testing, lay a solid foundation in effective disease management and modern healthcare strategies. In addition, advances in molecular diagnostic technologies have had a great impact on establishing the rapidly evolving pharmacogenomics field, resulting in the development of the era of personalized medicine and overall improvement in the healthcare system.^1^

DNA damage plays a significant role in cellular dysfunction and death. Defects in the DNA repair pathways result in genomic instability. In replicating cells, this could lead to cellular transformation, eventually leading to cancer development. On the other hand, in nonreplicating cells such as neuronal tissue, a consequence of loss of genomic integrity is apoptosis leading to neurodegenerative disorders such as Alzheimer’s disease and Parkinson’s disease.^2^,^3^ Topoisomerases (TOP) and tyrosyl DNA phosphodiesterases (TDP) are among the DNA repair players that play a fundamental role in regulating gene transcription, DNA replication, recombination, and repair through different mechanisms.^4^ Failure in the activities of these proteins results in protein-linked DNA breaks (PDBs), ultimately leading to neurodegenerative diseases and cancers.^5^,^6^

Patients with neurologic disorders such as intellectual disability, seizures, and ataxia have mutations in TDP2. As a result, TDP2 expression levels are affected, leading to abortive TOP2 activity and increased hypersensitivity to TOP2-induced double-stranded breaks.^7^ Moreover, TOP1 is inevitability required for proper synaptic function and regulates the levels of multiple synaptic proteins and, thereby, its dysfunction has a negative impact on the synaptic activity.^8^ In our recent publication, we have revealed that TOP1-mediated PDBs and R-loops lead to genomic instability in mice neurons, human cells, and in spinal cord tissues of patients suffering from amyotrophic lateral sclerosis and frontotemporal dementia.^9^ Furthermore, TDP1 expression was recently found to be decreased in spinocerebellar ataxia as a result of the downregulation UCHL3.^10^ Therefore, TOP1 and TDP2 transcripts level can be utilized as potential biomarkers for many neurodegenerative disorders.

On the other hand, TOP1 gene expression can be used as an early biomarker for predicting the response to TOP1-targeting chemotherapeutics.^4^ A significant correlation was found to exist in various colon and breast cancer cell lines between TOP1 expression and the sensitivity to SN-38, the active metabolite of the TOP1 poison, irinotecan.^11^–^13^ In addition, TDP2 is considered to be a potential biomarker of sensitivity to anticancer drugs such as etoposide, doxorubicin, and bicalutamide.^14^ TDP2 depletion in A549 and H460 lung cancer cell lines as well as chicken DT40 cells increased the sensitivity of the cells to etoposide.^5^,^15^,^16^ Moreover, mutant-p53-dependent overexpression of TDP2 has been implicated in cellular resistance to etoposide in lung cancer cells.^17^ Furthermore, we have recently shown that TDP1 expression is increased in rhabdomyosarcoma as a result of the upregulation of UCHL3.^10^ As a result, the expression levels of TOP1 and TDP2 can be measured and utilized as a potential biomarker in various cancers to predict and monitor patients’ response to different chemotherapeutics.

Sensitive and precise measurement of the mRNA transcripts expression level is of pivotal importance in enhancing our understanding to the cellular dogma, leading ultimately to accurate diagnosis and, hence, allowing physicians to make more informed clinical decisions. This opens the door for the development of more personalized therapeutic approaches, maximizing patients’ benefit and overcoming the side effects and drawbacks of the current conventional therapy.^18^

Among the tools used for relative and absolute quantification of mRNA are Northern blotting,^19^,^20^ RNA in situ hybridization,^21^,^22^ nuclease protection assays,^23^ and real-time PCR.^24^–^26^ Despite the high sensitivity and specificity of the aforementioned techniques, especially the latter, they are expensive, laborious, and require trained personnel and adequately equipped laboratories. In addition, mRNA absolute quantification is a multistep procedure that requires serially diluted standards to generate a standard curve. The standard could be double- or single-stranded DNA, or cRNA, which has the same sequence as the target RNA.^27^ Although DNA standards have the advantage of being more stable than RNA standards and exhibit a wider dynamic range, they cannot be used for one-step real-time PCR due to the lack of availability of a control that can measure the efficiency of the reverse transcriptase step. Therefore, this necessitates the need for alternative approaches for the absolute quantification of RNA with an acceptable sensitivity, specificity, and cost effectiveness.

In the last two decades, the unique physicochemical properties of gold nanoparticles (AuNPs) have been exploited considerably in the field of clinical diagnostics.^28^ The surface plasmon resonance (SPR) phenomenon of AuNPs is responsible for their intense colors, and large absorption and cross-sectional scattering properties.^29^ As a result, several colorimetric AuNPs-based detection methods have been developed.^30^–^34^ In our recent publication, we have developed a gold aggregating gold (GAG) biosensor to detect and quantify the hepatitis C virus RNA in clinical serum samples, as an alternative approach to the real-time PCR.^35^

**Figure 1:**
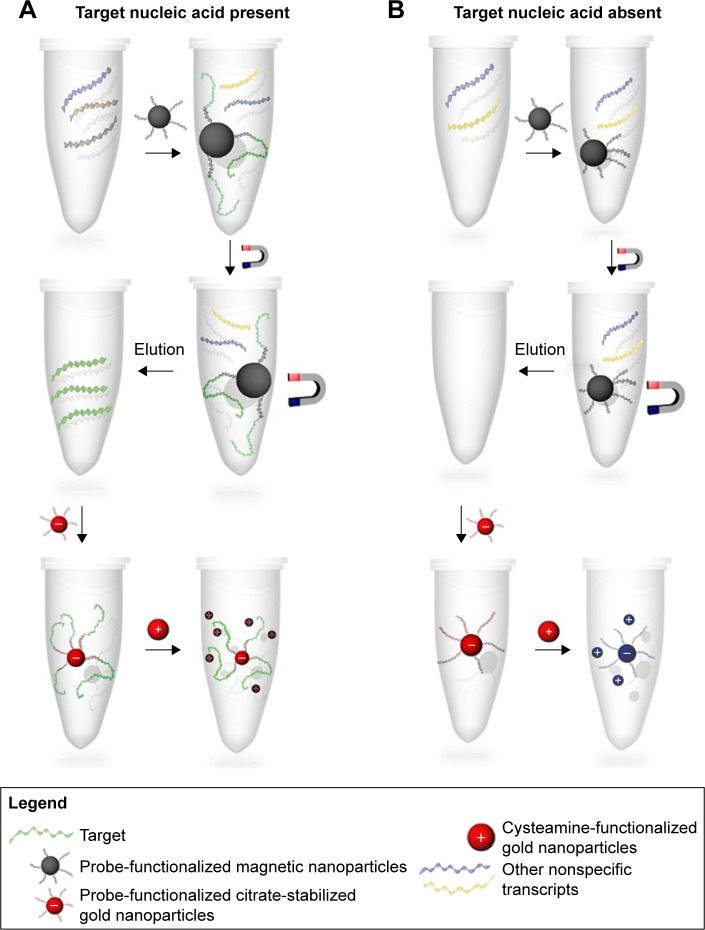
A scheme depicting the steps of specific RNA extraction using MNPs and detection using gold aggregating gold method. **Notes:** MNPs functionalized with target-specific probe are mixed with the total RNA to specifically capture the target RNA. (**A**) In the presence of the target mRNA, it is captured with the probe-functionalized MNPs and after washing and elution, the probe-functionalized citrate-stabilized AuNPs were added. After hybridization takes place by sequence complementarity between the probe and the target mRNA, cysteamine-functionalized AuNPs are added. The mixture color remains red in color, reflecting the dispersion of the AuNPs onto the target mRNA indicating a positive result. (**B**) In the absence of the target mRNA, addition of MNPs functionalized with the target RNA probe reveals no mRNA after elution. Consequently, no hybridization takes place with the probe-functionalized citrate-stabilized AuNPs and, hence, the addition of cysteamine-functionalized AuNPs results in a change of the solution color from red to blue. This is due to the interaction of the cysteamine AuNPs with the probe’s phosphate backbone electrostatically, thereby reducing the interparticle distance between the probe-functionalized citrate-stabilized AuNPs and the cysteamine-functionalized AuNPs, resulting in their aggregation and indicating a negative result. **Abbreviations:** AuNPs, gold nanoparticles; MNPs, magnetic nanoparticles.

Herein, we have exploited the optical properties of the AuNPs for the first time for the absolute quantification of unamplified mRNA transcripts using our developed GAG approach. TOP1 and TDP2 transcripts from cell lines have been extracted and quantified by copy number as well as confirmed and validated with qPCR and have demonstrated comparable sensitivity and specificity. Our data show that the GAG method (Figure 1) could be used efficiently in capturing and quantifying RNA of any origin with high specificity, sensitivity, and reproducibility.

## Materials and methods

### Synthesis and characterization of magnetic nanoparticles (MNPs)

MNPs were prepared by the chemical coprecipitation method as described previously with slight modifications.^36^,^37^ Briefly, ferrous and ferric chloride in a 1:2 molar ratio were mixed with deionized water purged with nitrogen gas for 30 minutes at 80°C. Then, 28% ammonium hydroxide solution was added dropwise to the above mixture under mechanical stirring. A black color developed, indicating the formation of the magnetite nanoparticles. Stirring and heating were continued till the pH was stable at 10. The resulting particles were washed thoroughly with 70% ethanol and deionized water at least thrice. The particles were then removed by magnetic decantation and dried in an oven overnight at 50°C. The MNPs were then characterized by high-resolution transmission electron microscopy (HR-TEM, JEOL-2100) and Fourier-transform infrared spectroscopy (Thermo Scientific Nicolet is-10).

### Synthesis and characterization of amino-functionalized MNPs

Synthesized MNPs were functionalized with aminopropyl triethoxy silane (APTES) according to Liu et al^38^ with slight modifications. Briefly, 500 mg of MNPs was dispersed in 100 mL nitrogen-purged deionized water by sonication, and then 2 mL of APTES was added to the above mixture. The reaction mixture was heated to 80°C, and stirred mechanically for 24 hours. After the reaction was completed, the black powder was separated by magnetic decantation, washed thoroughly with 70% ethanol and deionized water thrice, and then dried in an oven overnight at 50°C. The MNPs-APTES were then characterized by HR-TEM (JEOL-2100) and Fourier-transform infrared spectroscopy (Thermo Scientific Nicolet is-10).

### Functionalization of MNPs with target-specific probes

Conjugation of TOP1- and TDP2-specific probes to the amino-functionalized MNPs was performed using a hetero-bifunctional cross-linker, 3-maleimidobenzoic acid N-hydroxyl succinimide (MBS). MBS has an NHS ester at one end that reacts with the primary amine groups forming a stable amide bond and a maleimide group at the other end, which reacts with the sulfhydryl groups forming a stable thioether linkage. The MNPs-APTES were washed twice with dimethyl sulfoxide (DMSO), then MBS dissolved in DMSO was added to the nanoparticles and mixed on a roller shaker for 1 hour at room temperature. The nanoparticles were then washed twice with DMSO followed by coupling buffer (0.1 M phosphate buffer and 0.1 M NaCl, pH 7) twice, and collected by a magnet. The particles were resuspended in the coupling buffer and the thiol-modified probes were added to the suspended particles and allowed to react on a roller shaker overnight at room temperature. Finally, the solution was removed by magnetic decantation and the MNPs functionalized with target-specific probes were resuspended in storage buffer (0.01 M phosphate buffer, 0.1 M NaCl, pH 7.4).

### Synthesis and characterization of citrate-capped AuNPs

Citrate-capped AuNPs were synthesized according to the well-established sodium citrate reduction method of gold (III) chloride.^39^–^41^ The AuNPs were characterized using HR-TEM (JEOL-2100), UV-Vis spectroscopy (Plate Reader, firm version 1.32, software version 3.00R2 and serial no 415-1587; BMG Labtech, Ortenberg, Germany), and dynamic light scattering (Zetasizer Nano ZS90; Malvern Instruments, Malvern UK).

### Functionalization of the citrate-capped AuNPs with target-specific thiolated probes (nanoprobe)

TOP1- and TDP2-specific probes were alkanethiol modified at their 5′ terminus. The probe sequence was complementary to the target mRNA. The sequence for TOP1 probe was 5′AGTCTTCTCGATACTGGTTCCG′3, whereas for TDP2 probe it was 5′CTAAGTAGGAACACACCCCTC′3. Citrate AuNPs were functionalized with each alkanethiol-modified mRNA target-specific probe using the salt-aging process.^42^ Briefly, 5 nmol of the alkanethiol probe was first lyophilized, and then, the disulfide cleavage of the probe was achieved by resuspension in 100 µL of 0.1 M dithiothreitol (Sigma Aldrich), dissolved in 0.17M phosphate buffer (pH 8), for 2–3 hours with occasional vortexing, while being wrapped in foil. The cleaved probes were then purified using NAP-5 columns (GE Healthcare Illustra) according to the manufacturer’s instructions. The concentration of the probes after cleavage was measured using Beer–Lambert law from their absorbance at 260 nm. Freshly cleaved probes were then added to the AuNP solution with the ratio of ~1 OD/mL nanoparticles. After 20 minutes incubation at room temperature, phosphate buffer and sodium dodecyl sulfate were added to a final concentration of 0.01M and 0.1%, respectively. Then, salting process was done by adding three portions of sodium chloride solution at 30 minutes intervals to bring the final concentration of sodium chloride to 0.3M. The solution was incubated overnight with very gentle shaking. Centrifugation (14,000 rpm for 30 minutes) was performed five times to wash and remove the excess probes. The concentration of the unconjugated probes was measured in the supernatant to calculate their number. The gold pellet was resuspended in 3 mL of assay buffer (0.01M phosphate, 0.15M NaCl, and 0.1% SDS, pH 7.4). The color of the solution containing the functionalized AuNPs was not different from the original AuNPs and no aggregation was observed, indicating the efficiency of the conjugation process. The probe-functionalized AuNPs were characterized by UV-Vis spectroscopy (Plate Reader BMG Lab tech, firm version 1.32, software version 3.00R2 and serial no 415-1587).

### Synthesis and characterization of cysteamine-functionalized AuNPs

Cysteamine AuNPs were prepared according to Kim et al^43^ with minor modifications. Four hundred microliters of 0.15M cysteamine solution was added to 30 mL of 0.001M HAuCl_4_⋅H_2_O and stirred for 20 minutes. Then 20 µL of freshly prepared 0.01M NaBH_4_ solution was added under dark conditions, with vigorous stirring, twice, with 20-minute intervals. The solution was left to stir vigorously in the dark for 6 hours until AuNPs were formed, which were then stored at 4°C. The formed nanoparticles were then characterized using HR-TEM (JEOL-2100), UV-Vis spectroscopy (Plate Reader BMG Lab tech, firm version 1.32, software version 3.00R2 and serial no 415-1587), and dynamic light scattering (Zetasizer Nano ZS90, Malvern Instrument Ltd. UK).

### RKOs and mouse embryonic fibroblast (MEF) cell lines propagation

RKOs cancer cell lines (*Homo sapiens*, tissue: colon, carcinoma) were purchased from ATCC, LGC Standards, Middlesex, UK.^44^ They were cultured at 37°C and 5% CO_2_ in RPMI-1640 medium, supplemented with 10% FBS, 1% l-glutamine, and 1% penicillin/streptomycin. TDP2 knockout mice and MEF were generated, maintained, and genotyped as described previously in our works^15^,^45^,^46^ in an outbreed mixed 129 Ola and C57BL/6 background. The use of these cell lines was approved by the Institutional Review Board at the University of Sussex as per previous publications and subsequently by the Center of Genomics, Zewail City of Science and Technology Committee.

### Total RNA and specific transcripts extraction

RNeasy mini kit (Qiagen, Cat No: 74106) was used for total RNA extraction following manufacturer’s instructions and then each transcript was further purified by each target-specific probe-functionalized MNPs. Approximately 100 ng of the extracted RNA was denatured by heating at 72°C for 2 minutes and then mixed with the functionalized MNPs at 1,000 rpm for 15–20 minutes at 60°C. The MNPs were then washed with assay buffer (0.01M phosphate buffer, 0.1 M NaCl, and 0.01% SDS, pH 7.4) which was preheated at 50°C and collected at the bottom of the tube by the action of a permanent external magnet. This washing step was repeated for three times. Elution of the desired target mRNA was done by adding deionized water, and heating at 72°C for 2 minutes then rapidly collecting the MNPs by an external magnet. The supernatant containing the target mRNA was collected and preserved at −80°C.

### GAG assay for mRNA transcripts detection and quantification

The assay was performed by mixing 5 µL of the target-specific nanoprobe with 10 µL of the mRNA sample and heating at 95°C for 3 minutes followed by incubating at room temperature for 5 minutes. Ten microliters of 1 nM cysteamine-functionalized AuNPs was then added to the solution and mixed well. The solution color was developed immediately and observed by naked eye while mixing. The color was stable for ~30 minutes, depending on the RNA concentration. The solutions were scanned spectrophotometrically from 400 to 750 nm (Plate Reader BMG Lab tech, firm version 1.32, software version 3.00R2 and serial no 415-1587).

### Quantification of TOP1 and TDP2 mRNA transcripts using real-time PCR

A standard curve was performed using serial dilutions of the amplicons generated from the mRNA extracted by the MNPs for each target. After mRNA extraction, 4 µL of the mRNA was taken for the cDNA reaction (total reaction volume 40 µL) using hexamer primer by iScript cDNA synthesis kit (Biorad, Cat No: 170–8891) according to the manufacturer’s instructions. Then, 2 µL of the cDNA reaction was taken for the qPCR reaction (QuantStudio Real-Time qPCR) for both TOP1 and TDP2 in two different reactions (10 µL total reaction volume). Sequences for the primers and details of the thermal cycler are described in the supporting information.

### Quantification of TOP1 and TDP2 mRNA transcripts using GAG method

Quantification of the targets’ transcripts using the GAG method was performed by preparing serial dilutions of each transcript concentration (10^1^–10^4^ copies/reaction volume), and quantified using the GAG assay. The spectral absorbance for each concentration was scanned spectrophotometrically in duplicate, and the ratio of the nonaggregated nanoparticles at wavelength (λ) of 530 nm to the aggregated nanoparticles at λ of 620 nm (A_530_/A_620_) was recorded and used to generate the standard curve, in which the A_530_/A_620_ ratio was plotted against the log RNA concentration.

## Results and discussion

### Synthesis, functionalization, and characterization of MNPs

**Figure 2:**
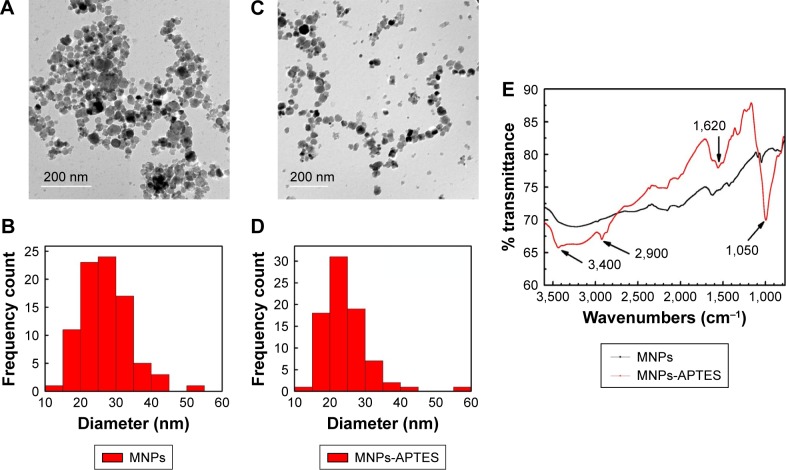
Characterization of the MNPs and MNPs-APTES using TEM and FTIR. **Notes:** (**A**) TEM images of magnetic nanoparticles. (**B**) Size distribution analyzed from TEM images of MNPs. (**C**) TEM images of APTES-functionalized MNPs. (**D**) Size distribution analyzed from TEM images of APTES-functionalized MNPs. (**E**) The FTIR spectra of as-synthesized MNPs and MNPs-APTES. **Abbreviations:** APTES, aminopropyl triethoxy silane; FTIR, Fourier transform infrared; MNPs, magnetic nanoparticles; TEM, transmission electron microscopy.

The MNPs were synthesized according to the coprecipitation method and were then functionalized with an amino functional group through silanization with 3-APTES. Functionalization of MNPs with amino groups was confirmed by their Fourier transform infrared spectra. The band at 1,050 cm^−1^ corresponds to the vibration of Si-O structure. The band at 2,900 cm^−1^ reveals the anchored propyl group of the APTES. The N-H bending and stretching vibration of the free amino groups is shown at 1,620 cm^−1^ and the characteristic fork-shaped band at 3,400 cm^−1^. In addition, the MNPs and MNPs-APTES were characterized by TEM, which showed that functionalization with APTES did not affect their average size and particles uniformity and homogeneity (Figure 2).

### Synthesis, functionalization, and characterization of AuNPs

**Figure 3:**
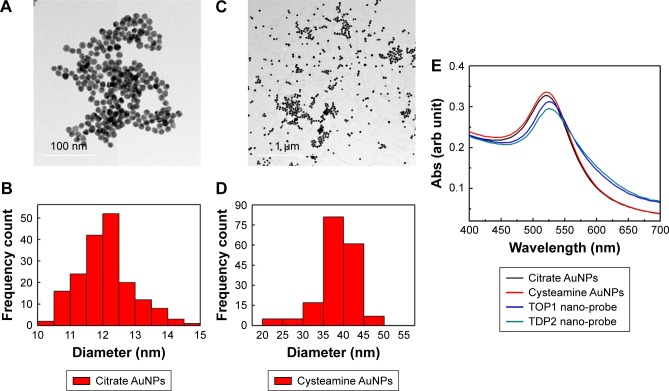
Characterization of the as-synthesized gold nanoparticles and the nanoprobes using TEM and UV-Vis spectrophotometer. **Notes:** (**A**) TEM images of citrate-capped AuNPs. (**B**) Size distribution analyzed from TEM images of citrate-capped AuNPs showing an average diameter of 12 nm. (**C**) TEM images of cysteamine-functionalized AuNPs. (**D**) Size distribution analyzed from TEM images of cysteamine-functionalized AuNPs showing an average diameter of 40 nm. (**E**) The extinction spectra of the as-synthesized citrate and cysteamine AuNPs showing λmax at 520 and 527 nm, respectively. TOP1 and TDP2 nanoprobes showed a slight red shift to 530 nm with a reduction in peak intensity and an increase in absorption above 600 nm confirming proper functionalization. **Abbreviations:** AuNP, gold nanoparticle; TDP2, tyrosyl DNA phosphodiesterase 2; TEM, transmission electron microscopy; TOP1, topoisomerase 1.

TEM analysis revealed that the citrate-capped and cysteamine-functionalized AuNPs were spherical in shape and uniformly distributed. The images were then analyzed using Image 1.41J software package (Wayne Rasband, National Institutes of Health USA. http://rsb.info.nih.gov/ij/Java1.6.0_05), to determine the size distribution of the particles. The citrate-capped and cysteamine-functionalized AuNPs had diameters of 12±2 and 40±5 nm, respectively (Figure 3). Zeta potential of the citrate-capped and the cysteamine-functionalized AuNPs were found to be -45.2 and +35.5 mV, respectively (Figure S1).

TOP1 and TDP2 transcript-specific nanoprobes were synthesized by functionalizing the citrate AuNPs with the alkanethiol-modified probes in two separate tubes, utilizing the advantage of the formation of a strong covalent bond between the thiol functional group and the gold surface.^47^ The synthesized nanoprobes were stable against low salt concentration and/or positively charged cysteamine-functionalized AuNPs induced aggregation. The UV-Vis spectrum of the as-synthesized citrate AuNPs revealed a λ_max_ at 520 nm, which was red-shifted to 530 nm after being functionalized with the target-specific probes. Furthermore, the cysteamine AuNPs revealed a λ_max_ at 527 nm (Figure 3E). Comparing the spectrum of the citrate and cysteamine AuNPs against the nanoprobes spectrum in Figure 3E, it is obvious that the aggregated populations of the AuNPs in the nanoprobes extinction spectra are slightly higher than the as-synthesized citrate AuNPs, confirming the increase in size of the citrate AuNPs after functionalization with the probes. The molar concentration of the citrate and cysteamine AuNPs was calculated according to Liu et al.^48^ to be about 3.5 and 1 nM, respectively. The amount of probes conjugated to the citrate AuNPs was calculated according to Hill and Mirkin^39^ and Hurst et al^42^ to be about 1.2 and 1.3 nmol for the TOP1 and TDP2 nanoprobes, respectively. Accordingly, about 130 and 115 probes of TDP2 and TOP1, respectively, were conjugated per nanoparticle. This slight difference between the two probes in terms of the conjugation amount has shown no significant effect on the assay procedures and quantification as discussed later.

**Figure 4:**
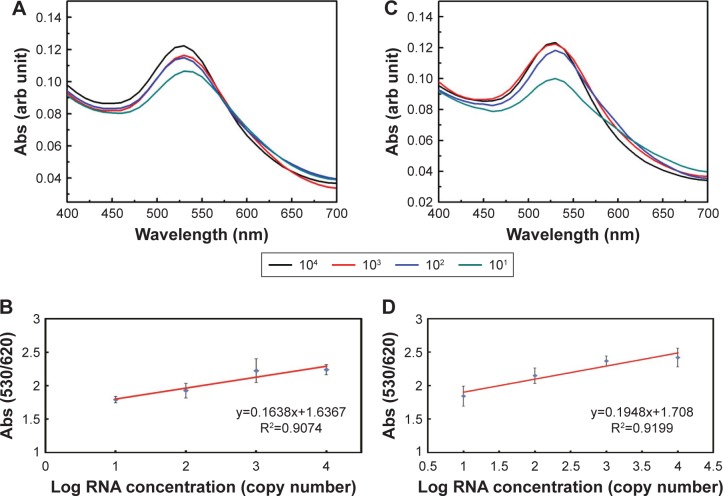
GAG assay spectrum results with TOP1 and TDP2 mRNA transcripts. **Notes:** (**A**) Different concentrations of TOP1 mRNA were detected and quantified by a specific probe using the GAG assay. The mRNA was extracted and then targeted by the conjugated nanoparticles. Different SPRs were obtained according to the TOP1 mRNA concentration (copy number). The aggregation behavior was compatible with both SPR and concentration. (**B**) A standard curve was performed by getting the absorbance ratio (A_530_/A_620_) against the log of the mRNA concentration. A linear relationship was obtained with R^2^=0.9074. (**C**) Different concentrations of TDP2 mRNA were targeted by a TDP2-specific probe using the GAG assay according to the TDP2 mRNA concentration (copy number). The aggregation behavior was compatible with both SPR and concentration. (**D**) A standard curve was performed by plotting the absorbance ratio (A_530_/A_620_) against the log of the mRNA concentration and a linear relationship was obtained with R^2^=0.9199. **Abbreviations:** GAG, gold aggregating gold; SPR, surface plasmon resonance; TDP2, tyrosyl DNA phosphodiesterase 2; TOP1, topoisomerase 1.

**Figure 5:**
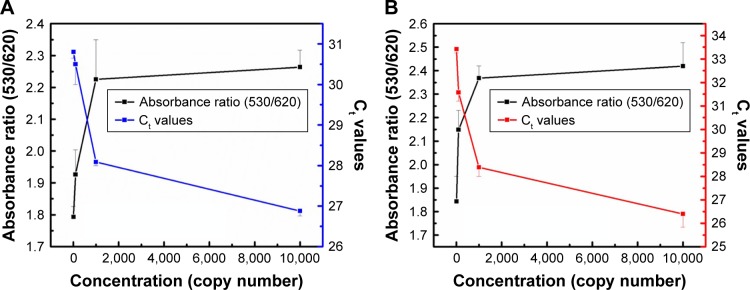
GAG assay vs the C_t_ of real-time PCR according to the copy numbers of (**A**) TOP1 mRNA and (**B**) TDP2 mRNA in RKO cell lines. The absorbance ratio (A_530_/A_620_) and the C_t_ were plotted vs the mRNA concentration (copy number). For both transcripts, the relation between the mRNA concentration vs both the C_t_ and the absorbance ratio is inversely proportional, which confirms the specificity and precision of the GAG nanoassay and its capability in competing with the real-time PCR for RNA absolute quantification. Increasing RNA concentration results in decreased C_t_ and increased absorbance ratio and vice versa. The error bars represent the standard error of each point. **Abbreviations:** C_t_, threshold cycle; GAG, gold aggregating gold; TDP2, tyrosyl DNA phosphodiesterase 2.

Quantification using the GAG approach of endogenous transcripts is quite challenging, as the detection and quantification is based on a probe of 22 bases long, which may increase the probabilities of false positive results. Therefore, MNPs functionalized with a target-specific probe have been employed herein for enrichment of the RNA pool with the target mRNA to minimize false positives and ensure the accuracy and the precision of the assay. After magnetic extraction of TOP1 and TDP2 transcripts from the human colorectal cancer RKO cell lines, a portion of each transcript was quantified using the absolute standard curve generated by the real-time PCR. Then, serial dilutions of the RNA were conducted and measured using the GAG assay. Our data indicate a linear correlation between the ratio of absorbance at wavelength 530 nm/absorbance at wavelength 620 nm for the target’s concentrations between 10^1^ and 10^4^ copies per reaction for both TOP1 and TDP2 (Figure 4). These data have been produced blindly at least twice by two independent researchers. The data confirm the reproducibility, sensitivity, and specificity of the GAG assay in the detection and quantification of any RNA using specific probes. The detection limit of the assay has been found to be ten copies per reaction. Linear association has been found between the GAG method and the real-time PCR for TOP1 and TDP2 detection/quantification in the range of 10^1^–10^4^ copies (Figure 5).

The melting curves confirming the efficiency and specificity at amplification and the standard curves generated for the TOP1 and TDP2 transcripts are shown in Figures S2–S4.

### Absorption performance of MNPs toward targeted mRNA transcripts

The total RNA extract from the cell lines was subjected to MNPs-based extraction and the transcript of interest was enriched through utilizing the MNPs functionalized with the target-specific probe. To investigate the capacity of the MNPs in capturing the mRNA of interest, the absorbance at 260 nm of the total RNA extract, as well as that found in the supernatant, washes, and the final elution after subjection to the MNPs was measured. The total amount of RNA that was found in the final elution was ten times lower than that in the starting total RNA extract. This is expected because the MNPs functionalized with the target-specific probe selectively capture the transcript of interest mainly.

Furthermore, RT-PCR was also conducted on the aforementioned total RNA, supernatant, washes, and the final elute for four mRNA transcripts: TOP1 and TDP2 as well as TOP2 and TDP1, which are structurally and functionally related to both TOP1 and TDP2. Although the final elute contains all the four transcripts, the transcript of interest captured by the MNPs functionalized with the target-specific probe was present in the highest amount. This confirms that the extraction using the MNPs functionalized with the target-specific probe enriches the presence of the target in the final elution. Moreover, the loss of the transcript of interest starts from the first supernatant (after incubation of the MNPs with the total RNA) to the final elute and is for about 20%. This confirms that most of the targeted mRNA transcripts captured by the MNPs are retained until elution.

### GAG assay specificity and reproducibility validation

**Figure 6:**
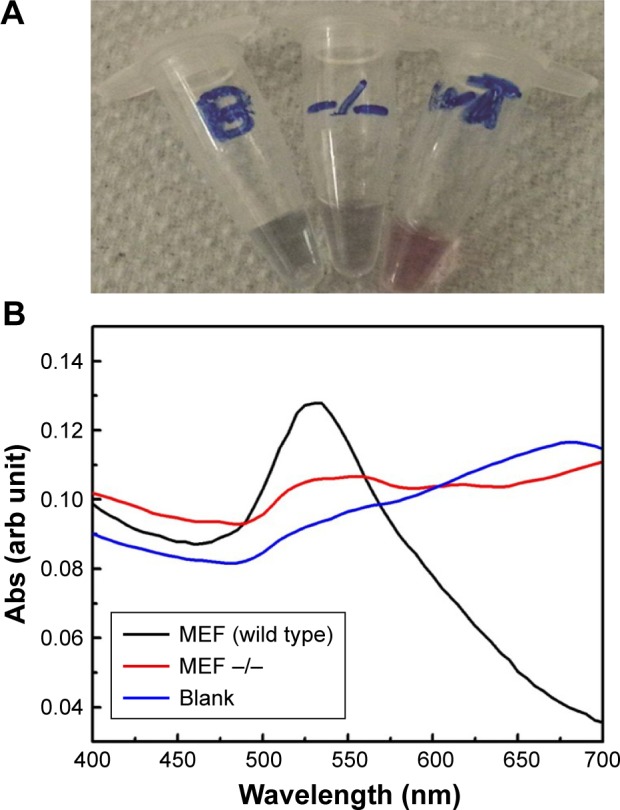
Detection of TDP2 in MEF cells using gold aggregating gold method. **Notes:** (**A**) A photograph of the final results of the GAG assay conducted on MEF wild-type and TDP2-deficient cell lines as well as a blank (negative control). The results show that the AuNPs have aggregated and turned blue in both the blank and Tdp2^−/−^ MEF cells while there was no aggregation observed in the wild-type MEF cells, with the AuNPs remaining red in color, indicating the presence of the TDP2 mRNA transcript. (**B**) The extinction spectra of the AuNPs after conducting the GAG assay on the blank, wild-type, and Tdp2^−/−^ MEF cells, confirming the nonaggregation of the AuNPs in the wild type due to the presence of TDP2 and the aggregation of the AuNPs in the blank and Tdp2^−/−^ due to the absence of TDP2. **Abbreviations:** AuNP, gold nanoparticle; GAG, gold aggregating gold; MEF, mouse embryonic fibroblast; TDP2, tyrosyl DNA phosphodiesterase 2.

To confirm the specificity and reproducibility of the GAG assay in distinguishing between different transcripts, the assay has been performed on two MEF cell lines, the wild-type and TDP2 knockout cells (Tdp2^−/−^ MEF). Total RNA was extracted from the two cell lines in parallel, and then extracted using MNPs functionalized with TDP2-specific probe. The assay was conducted on the magnetic extracted RNA. Positive results were revealed by the GAG method for the wild-type MEF cell lines and negative for the Tdp2^−/−^ MEF cell lines. The extinction spectra and the assay colors of the wild-type and Tdp2^−/−^ MEFs are shown in Figure 6. Further confirmation was obtained by repeating the assay at three different passages using three independent biologic replicates.

### Mechanism of GAG assay

After eluting the RNA transcript, it is added to the nanoprobe and denatured at 95°C for 3 minutes. This is necessary to allow the accessibility of the probes to the RNA molecules, through unfolding the RNA tertiary structure. As a result, hybridization between the target mRNA and the complementary probe occurs and when the mixture is subsequently cooled to room temperature, the RNA would then refold to adopt a more favorable, thermodynamically stable form. The folding of the target mRNA around the specific nanoprobe stabilizes it against the aggregation induced by the addition of the cysteamine-functionalized AuNPs as it results in the formation of multiple layers of RNA. Cysteamine-functionalized AuNPs are distributed along the RNA molecules, with their positively charged amino groups interacting electrostatically with the negatively charged phosphate backbone of the target RNA shielding the nanoprobe, hence preventing its aggregation. In the absence of the target RNA, the positively charged cysteamine-functionalized AuNPs would interact with the negatively charged phosphate backbone of the probe functionalized on the AuNPs (nanoprobe), which would bring them in close proximity, resulting in the aggregation of the AuNPs.

## Conclusion

In conclusion, we demonstrated the potential of the GAG assay as a biosensor designed to specifically detect and quantitate endogenous genome stability transcripts that are clinically important for both cancer and neurologic disease. Our method is useful for diagnosing and monitoring the progression of disease and can be used as an indicator for response to chemotherapeutic treatment. The developed biosensor could compete with the real-time PCR in measuring gene expression with a detection limit of ten copies of the specific mRNA target. Moreover, the biosensor has the potential in discriminating between the target mRNA in cells expressing or not expressing the desired target. The overall time of the assay is ~30–45 minutes including the mRNA extraction. Moreover, absolute quantification of RNA has been achieved without the tedious and labor-intensive real-time PCR standard curve generation of the RNA target. Therefore, the developed biosensor could be used in small laboratories in rural areas where sophisticated equipment and well-trained personnel are not available.

## Supplementary materials

### Molar concentration calculations

Molar concentration of the citrate-capped and cysteamine-functionalized AuNPs was calculated using equations A.1 and A.2, assuming that all the gold ions have been consumed in the reaction and the particles are spherical in shape forming face-centered cubic (FCC) phase within the AuNPs.^1^

$$N=\frac{\pi }{6}\frac{\rho D^{3}}{M}=30.89602D^{3}$$ (A.1)

where *N* is the average number of gold atoms per particle, *ρ* is the density for FCC gold (19.3 g/cm^3^), *M* is the atomic weight of gold (197 g/mol), and *D* is the average diameter, in nanometer, for the AuNPs as analyzed from the TEM images.

Then, the molar concentration is calculated according to the following equation:

$$C=\frac{N_{\text{total}}}{NVN_{A}}$$ (A.2)

in which *N*_total_ is the number of gold nanoparticles in the reaction volume, *N* is the average number of gold atoms calculated from equation A.1, whereas *V* is the volume of the reaction solution in liters, and *N*_A_ is Avogadro’s number.

### Characterization of citrate-capped and cysteamine-functionalized gold nanoparticles

Zeta potential of the synthesized AuNPs was recorded by zeta Sizer. Each sample was measured three times and scanned 100 times for each measurement, and both mean and SD for each sample was calculated. The zeta potential of citrate and cysteamine AuNPs is shown in Figure S1.

### Generation of absolute standard curves for quantification of TOP1 and TDP2 transcripts using real-time PCR

For TOP1, the forward primer was 5′GAAGTCCGGC ATGATAACAAGG′3, whereas the reverse primer was 5′AGTCTTCTCGATACTGGTTCCG′3. On the other hand, for TDP2, the forward primer was 5′AGCCC AAGACCTATGTTGACC′3, whereas the reverse primer was 5′CTAAGTAGGAACACACCCCTC′3. The annealing temperature was adjusted specifically for each transcript to be 57.3°C for TOP1 and 55.3°C for TDP2. The thermal cycler for TOP1 was 95°C for 15 seconds, 57.3°C for 60 seconds, and 95°C for 15 seconds for 35 cycles, whereas the thermal cycler for TDP2 was 95°C for 15 seconds, 55.3°C for 60 seconds, and 72°C for 20 seconds for 35 cycles.

All the reaction volume for both transcripts was taken and run on agarose gel, and the specified band was excised from the gel and purified by the QiAquick Gel Extraction Kit (Cat No 28706) according to the manufacturer’s instructions. Further purification was done for the amplicons using QiAquick PCR Purification Kit (Cat No 28104), according to the manufacturer’s instructions. These processes were repeated three times to get a high yield and concentration of the PCR product for both transcripts. The final amplicon concentrations were measured using UV at 260 nm, and the copy numbers were calculated according to the base pairs of each amplicon to be 187 and 183 for TOP1 and TDP2, respectively.

Serial dilutions of the purified amplicons were then performed for TOP1 and TDP2 (10^1^–10^10^), and qPCR with the same abovementioned cycling conditions was performed for each dilution and standard curves for TOP1 and TDP2 were generated as shown in Figures S3 and S4, respectively. These standard curves were then used for calculating the copy numbers of RNA in the nanoassay.

Figure S1 Zeta potential measurements. (**A**) Zeta potential of the citrate-capped gold nanoparticles (−45.2 mV). (**B**) Zeta potential of the cysteamine-functionalized gold nanoparticles (+35.5 mV).

Figure S2 TOP1 and TDP2 melting curves.
**Notes:** (**A**) The results showed one melting curve for the different serial dilutions of purified *TOP1* PCR product. (**B**) Different dilutions of TDP2 showed the same melting curve with different concentrations.
**Abbreviations:** TDP2, tyrosyl DNA phosphodiesterase 2; TOP1, topoisomerase 1.

Figure S3 TOP1 standard curve generated from the amplicons serial dilutions.
**Notes:** As shown, C_t_ decreases with increase in copy number, with a linear correlation between the C ^2^t and the concentration. R =0.973. The PCR efficiency was 115.626%.
**Abbreviation:** TOP1, topoisomerase 1.

Figure S4 TDP2 standard curve generated from the amplicons serial dilutions.
**Notes:** As shown, C_t_ decreases with increase in copy number, with a linear correlation between the C_t_ and the concentration. R^2^=0.995. The PCR efficiency was 99.905%.
**Abbreviation:** TDP2, tyrosyl DNA phosphodiesterase 2.

Reference1LiuXAtwaterMWangJHuoQExtinction coefficient of gold nanoparticles with different sizes and different capping ligandsColloids Surf B Biointerfaces2007581371699753610.1016/j.colsurfb.2006.08.005
