# Supplementary figures and images for: Gold nanoparticles – an optical biosensor for RNA quantification for cancer and neurologic disorders diagnosis
Source: Int J Nanomedicine. 2018 Nov 29;13:8137–51. doi: 10.2147/IJN.S181732 (PMC6278840; doi:10.2147/IJN.S181732)

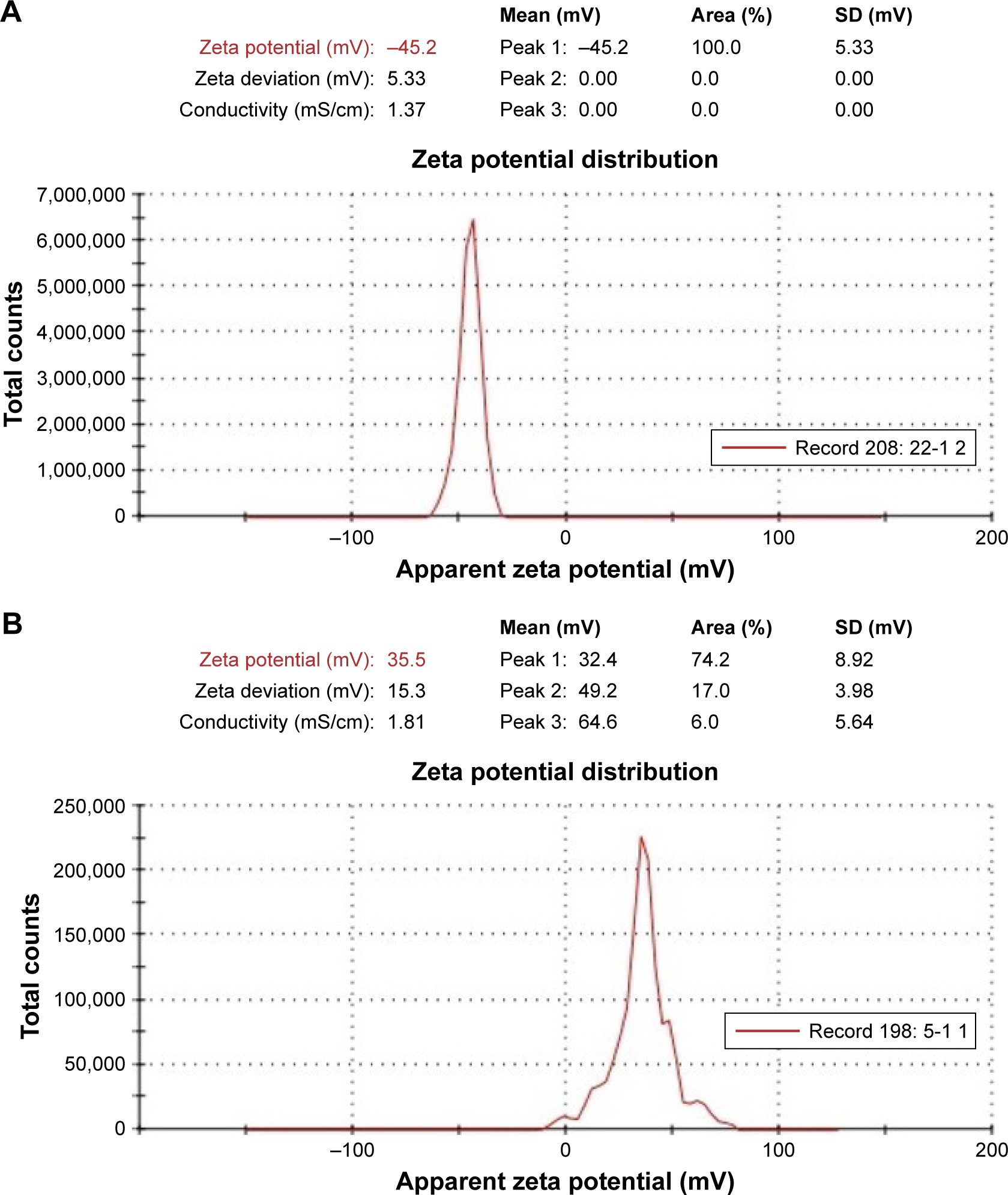

Supplement: Figure S1 — Zeta potential measurements. (A) Zeta potential of the citrate-capped gold nanoparticles (−45.2 mV). (B) Zeta potential of the cysteamine-functionalized gold nanoparticles (+35.5 mV). [file ijn-13-8137s1.tif]

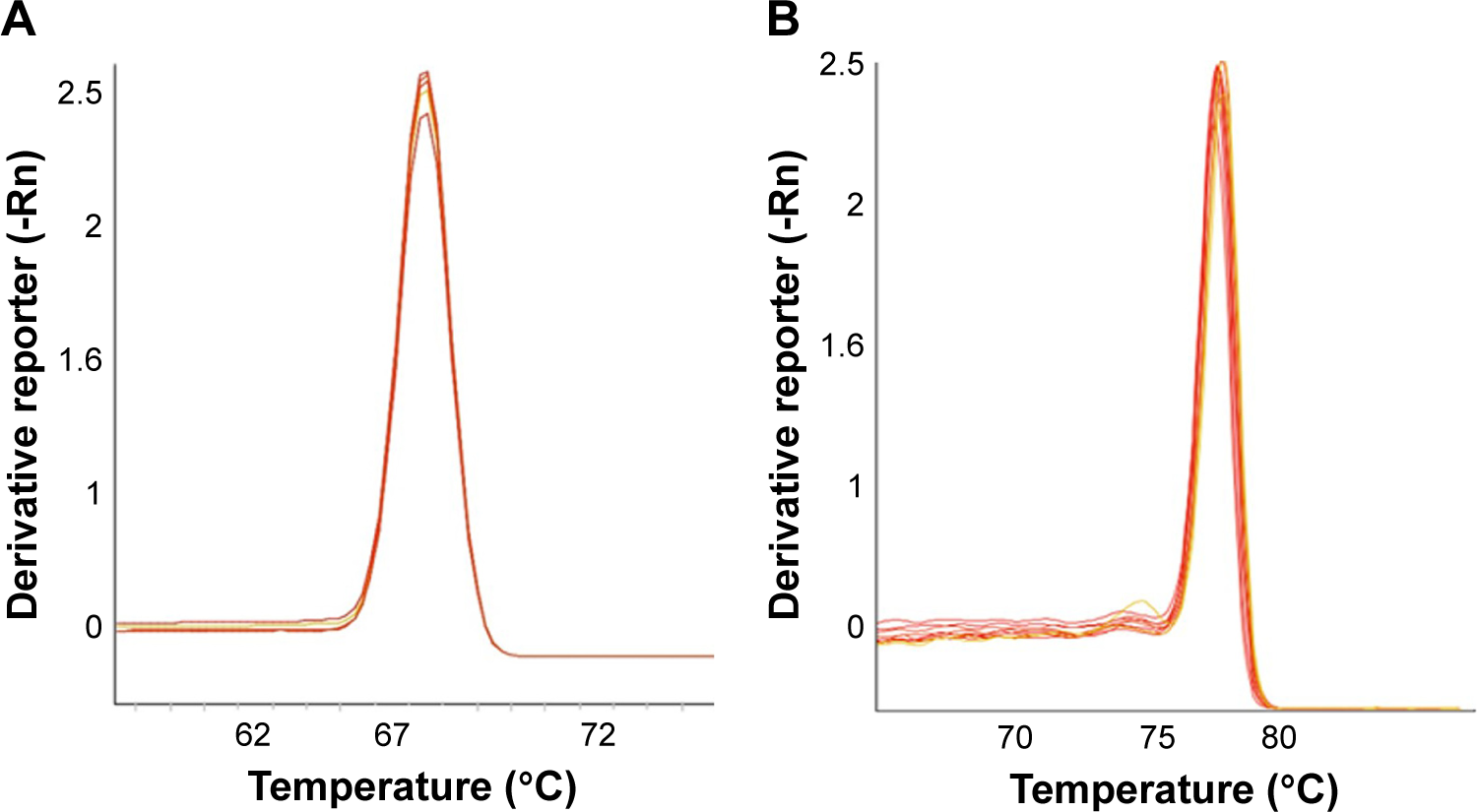

Supplement: Figure S2 — TOP1 and TDP2 melting curves. Notes: (A) The results showed one melting curve for the different serial dilutions of purified TOP1 PCR product. (B) Different dilutions of TDP2 showed the same melting curve with different concentrations. Abbreviations: TDP2, tyrosyl DNA phosphodiesterase 2; TOP1, topoisomerase 1. [file ijn-13-8137s2.tif]

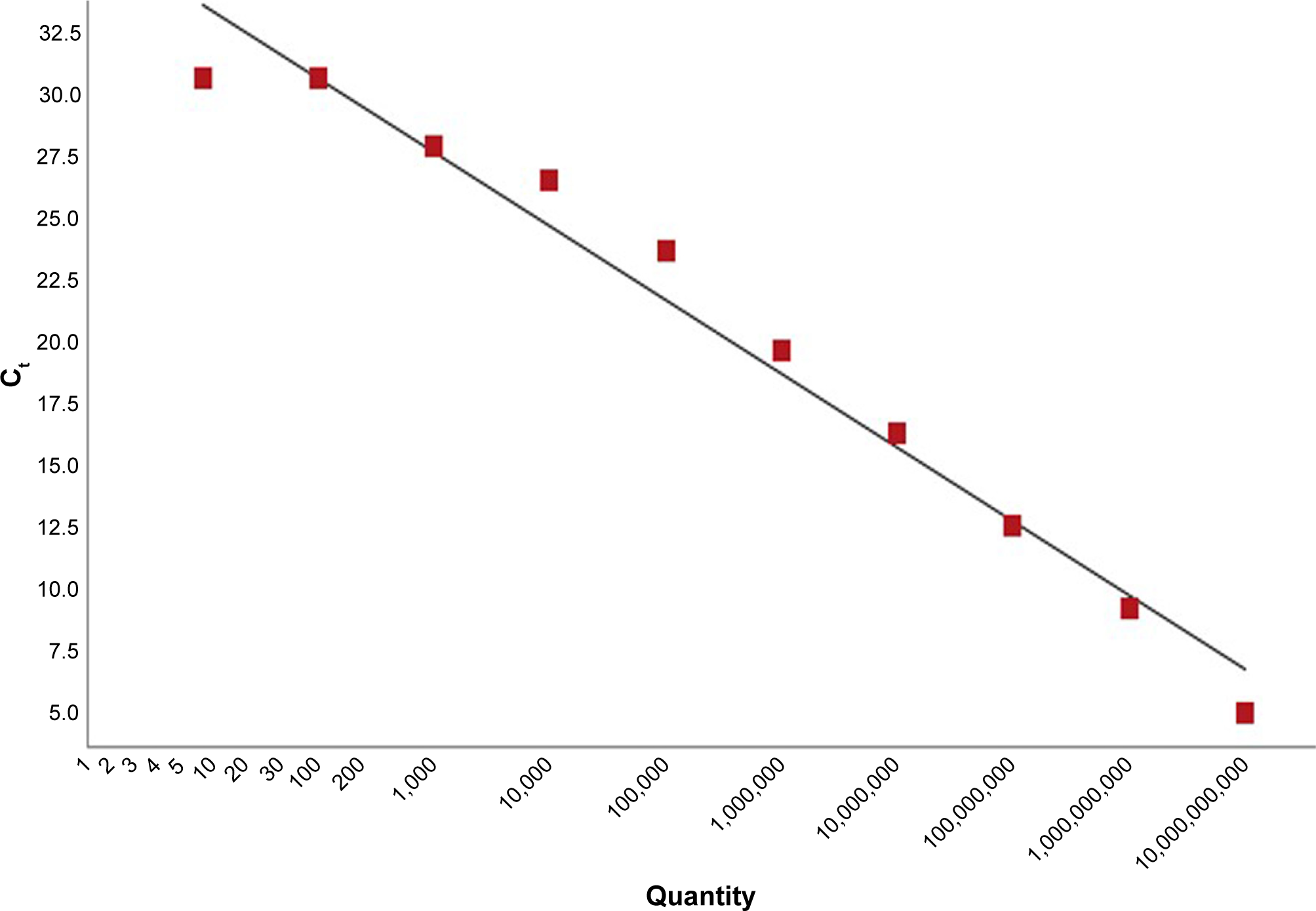

Supplement: Figure S3 — TOP1 standard curve generated from the amplicons serial dilutions. Notes: As shown, Ct decreases with increase in copy number, with a linear correlation between the C 2t and the concentration. R =0.973. The PCR efficiency was 115.626%. Abbreviation: TOP1, topoisomerase 1. [file ijn-13-8137s3.tif]

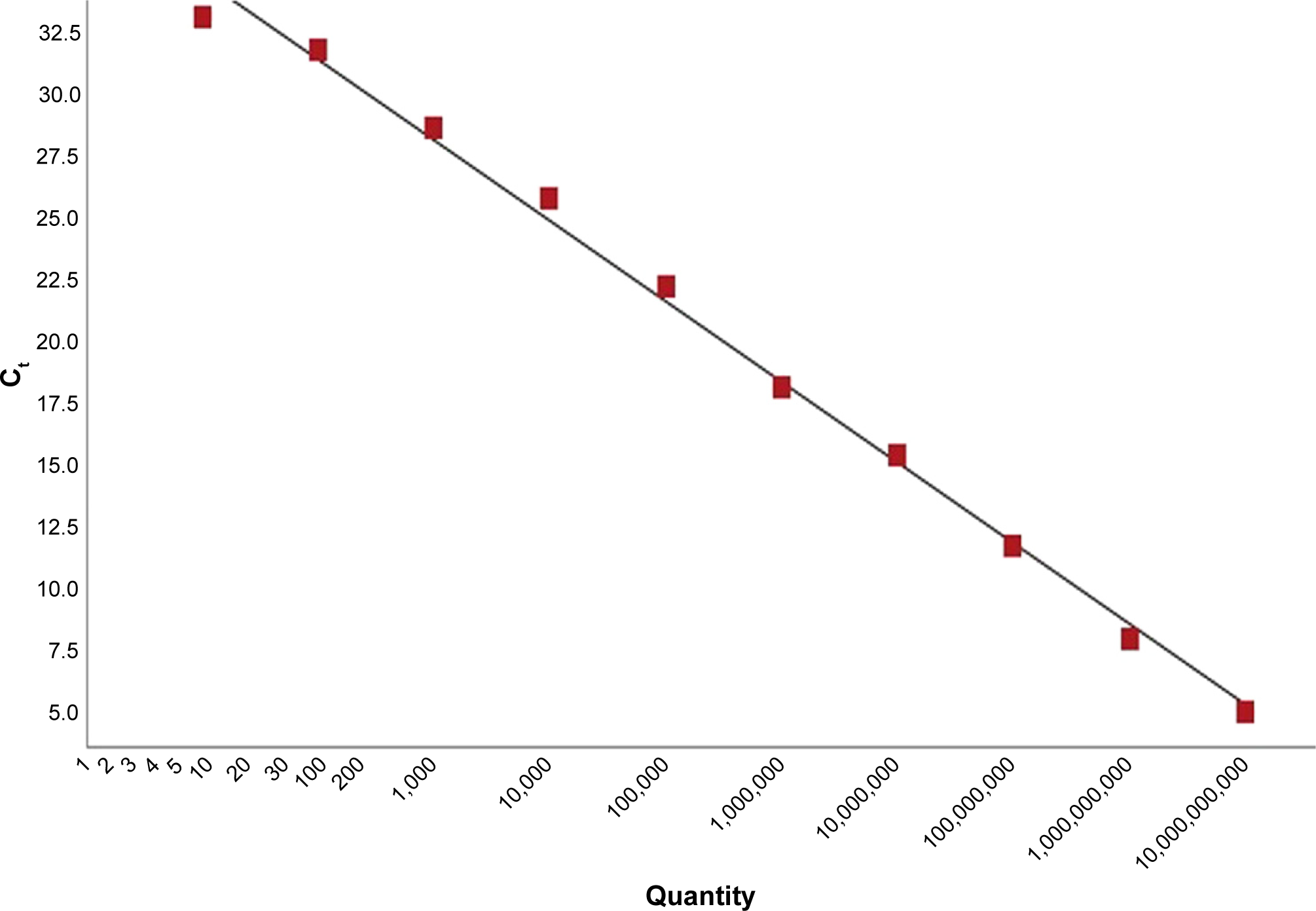

Supplement: Figure S4 — TDP2 standard curve generated from the amplicons serial dilutions. Notes: As shown, Ct decreases with increase in copy number, with a linear correlation between the Ct and the concentration. R2=0.995. The PCR efficiency was 99.905%. Abbreviation: TDP2, tyrosyl DNA phosphodiesterase 2. [file ijn-13-8137s4.tif]
